# Impact of the Mass Drug Administration for malaria in response to the Ebola outbreak in Sierra Leone

**DOI:** 10.1186/s12936-016-1493-1

**Published:** 2016-09-20

**Authors:** Maru Aregawi, Samuel J. Smith, Musa Sillah-Kanu, John Seppeh, Anitta R. Y. Kamara, Ryan O. Williams, John J. Aponte, Andrea Bosman, Pedro Alonso

**Affiliations:** 1 World Health Organization, Global Malaria Programme, Geneva, Switzerland; 2 Ministry of Health and Sanitation (MoHS), Freetown, Sierra Leone; 3 ISGlobal, Barcelona Ctr. Int. Health Res. (CRESIB), Hospital Clínic-Universitat de Barcelona, Barcelona, Spain

## Abstract

**Background:**

As emergency response to the Ebola epidemic, the Government of Sierra Leone and its partners implemented a large-scale Mass Drug Administration (MDA) with artesunate–amodiaquine (ASAQ) covering >2.7 million people in the districts hardest hit by Ebola during December 2014–January 2015. The World Health Organization (WHO) and the National Malaria Control Programme (NMCP) evaluated the impact of the MDA on malaria morbidity at health facilities and the number of Ebola alerts received at District Ebola Command Centres.

**Methods:**

The coverage of the two rounds of MDA with ASAQ was estimated by relating the number anti-malarial medicines distributed to the estimated resident population. Segmented time-series analysis was applied to weekly data collected from 49 primary health units (PHUs) and 11 hospitals performing malaria parasitological testing during the study period, to evaluate trends of malaria cases and Ebola alerts during the post-MDA weeks compared to the pre-MDA weeks in MDA- and non-MDA-cheifdoms.

**Results:**

After two rounds of the MDA, the number of suspected cases tested with rapid diagnostic test (RDT) decreased significantly by 43 % (95 % CI 38–48 %) at week 1 and remained low at week 2 and 3 post-first MDA and at week 1 and 3 post-second MDA; RDT positive cases decreased significantly by 47 % (41–52 %) at week 1 post-first and remained lower throughout all post-MDA weeks; and the RDT test positivity rate (TPR) declined by 35 % (32–38 %) at week 2 and stayed low throughout all post-MDA weeks. The total malaria (clinical + confirmed) cases decreased significantly by 45 % (39–52 %) at week 1 and were lower at week 2 and 3 post-first MDA; and week 1 post-second MDA. The proportion of confirmed malaria cases (out of all-outpatients) fell by 33 % (29–38 %) at week 1 post-first MDA and were lower during all post-MDA weeks. On the contrary, the non-malaria outpatient cases (cases due to other health conditions) either remained unchanged or fluctuated insignificantly. The Ebola alerts decreased by 30 % (13–46 %) at week 1 post-first MDA and much lower during all the weeks post–second MDA.

**Conclusions:**

The MDA achieved its goals of reducing malaria morbidity and febrile cases that would have been potentially diagnosed as suspected Ebola cases with increased risk of nosocomial infections. The intervention also helped reduce patient case-load to the severely strained health services at the peak of the Ebola outbreak and malaria transmission. As expected, the effect of the MDA waned in a matter of few weeks and malaria intensity returned to the pre-MDA levels. Nevertheless, the approach was an appropriate public health intervention in the context of the Ebola epidemic even in high malaria transmission areas of Sierra Leone.

**Electronic supplementary material:**

The online version of this article (doi:10.1186/s12936-016-1493-1) contains supplementary material, which is available to authorized users.

## Background

Malaria transmission in Sierra Leone, with a population of 6.3 million [1], is intense with little seasonal fluctuations. Recent surveys have documented high parasite prevalence (43 % in children under five) [2] and high under-five child mortality (156 per 1000 live birth) [3]. In 2013, prior to the Ebola viral disease (EVD) outbreak, the country scaled-up anti-malarial interventions and, as a result, 62 % of households owned at least one long-lasting insecticidal net (LLIN) and 39 % of the population slept under an LLIN the night before the survey; 37 % of children with fever took artemisinin-based combination therapy (ACT) [2]; and >85 % of the suspected cases in the public sector were parasitologically tested [4]. In June 2014, over 3.5 million LLINs were distributed targeting the entire population. In 2014, Sierra Leone became the worst affected country by the Ebola outbreak, with  >8704 confirmed cases; and >3589 deaths as of 3rd November 2015 when WHO certified the country Ebola-free [5].

At the same time malaria burden exerted a heavy toll to the health care system, which was compromised by the EVD epidemic due to high mortality to frontline health workers and closure of many facilities both in the public and private sectors. Fear of being referred to Ebola holding centres and of contracting EVD nosocomial infections significantly reduced patient attendance to health facilities by up to 40 % from May to September 2014, but the attendance returned to normal (only 7 % less) by December 2014 [6]. The similarities of the initial clinical presentations of EVD with that of malaria, i.e., fever, anorexia, fatigue, headache and joint pains posed a problem of differential diagnosis for both patients and health care workers. As a result, patients with symptoms of malaria had been shunning away from seeking care for fear of being suspected as EVD and referred to Ebola holding centres—leading to increased malaria morbidity and mortality for lack of prompt diagnosis and effective treatment. Recent estimates showed that absence of regular access to health care services during the Ebola epidemic, may have led to an increase of untreated malaria cases by 88 % (95 % CI 83–93) or 207 per 1000 population in Sierra Leone, equivalent to 1.3 million (0.9–1.9 million) untreated cases [7]. A similar estimate in Guinea showed increase in the number of malaria cases as a result of changes in health-seeking behaviour caused by the Ebola epidemic [8].

In response to the Ebola outbreak and its impact on the malaria burden, WHO issued an interim recommendation for malaria prevention and control in EVD disease-affected zones in November 2014. WHO recommended changes in testing practices promoting use of personal protective equipment in health facilities and “no touch” approach at community level, new approaches for LLINs distribution to avoid exposure to EVD due to overcrowding and MDA using ACT in areas heavily affected by Ebola, where malaria transmission is high and access to treatment is very low [9]. As an emergency response, the MoHS instructed MDA and presumptive treatment of all Ebola suspected cases in the Holding centres with ASAQ (Coarsucam™) without parasitological testing (Additional file 1). The objectives of the MDA were to rapidly reduce (i) malaria burden and (ii) the number of febrile cases which could be considered as suspected EVD cases and referred to Ebola Holding Centres, increasing the risk of nosocomial infection. The MDA did not aim for a long-term reduction of malaria transmission in the areas.

The NMCP of the Ministry of Health and Sanitation (MoHS), in collaboration with Médecins Sans Frontières (MSF) Spain, United Nations Children’s Fund (UNICEF), The Global Fund to Fight HIV/AIDs, Tuberculosis and Malaria (GFATM) and WHO led a large scale of MDA using ASAQ-the first-line antimalarial medicine in the country.

The MDA involved micro-planning at district level with participation of local authorities (paramount chiefs, councillors, primary health unit in-chiefs, regional and national supervisors); and major partners including MSF-Spain, UNICEF and WHO. Quantification of ASAQ needs was conducted based on the house-to-house population registration previously conducted for the LLIN mass campaign in 2014 using four age categories (2 % for 6–11 months; 13.7 % for 12–59 months; 28 % for 5–13 years; 54.3 % for 14 years and above). The two rounds of MDA targeted to cover at least 85 % of the three million people living in Ebola affected districts (with at least 85 % adherence to treatment) and were implemented at 5 weeks interval during, 5–8 December 2014 and 16–19 January 2015.

Funding for the procurement of six million ASAQ doses and operational costs was provided by partners. Quality control analysis of ASAQ samples was undertaken by the Pharmacy Board of Sierra Leone.

Administration of ASAQ was carried out in four regimens, door-to-door, with directly-observed treatment (DOT) for the first dose with counselling to complete the full 3-day treatment courses without supervision. The MDA campaign excluded: (1) children below 6 months, (2) malnourished children, (3) pregnant women in their first trimester; persons with fever or feeling unwell, (4) persons who received ASAQ within the last month, and (5) patients taking Zidovudine, Efavirenz or co-trimoxazole. Family members in quarantined households (with confirmed or suspected Ebola cases) were not visited by the MDA campaign but were counted and provided with ASAQ by the Ebola surveillance teams with personal protective equipment (PPE) following the standard safety procedures.

A total of 8330 health staff and community health workers (CHWs) were trained and deployed for the campaign, of which 5000 CHWs were in the capital, Western Area, alone. Distribution teams (each comprising a health worker from the nearest health facility and a CHW) visited at least 150 persons/day for 4 days. Supervision was conducted by 48 national and 833 district supervisors. In addition, 70 independent monitors visited 8400 households in 4 days and assessed the coverage and quality of the MDA during the campaign (In-process) and immediately after (End-process); and 33 health staff specifically trained in pharmacovigilance interviewed a total of about 19,000 persons after each cycle and recorded reports of adverse effects.

Intensive social mobilization before and during the MDA campaign included advocacy meetings with local stakeholders; press briefing, jingle slots on national radios and local FMs and TV panel discussions; posters and banners; megaphone-mounted vehicles and town criers. Key messaging focussed on benefits of ASAQ to reduce fever and how to avoid possible confusion with Ebola suspects; expected common side effects of ASAQ; importance of adherence to treatment; beneficiary and excluded groups; and what to do and where to report in event of suspected adverse drug reaction.

Given the scope of the MDA as emergency measure to reduce the burden of malaria in the context of the Ebola outbreak, its unprecedented large scale in Africa, and the significant investments made, WHO and NMCP collaborated to evaluate the impact of the MDA on malaria morbidity and burden of cases presenting as Ebola suspected patients.

This study aimed to primarily assess the impact of the MDA on trends of number of malaria cases attending health facilities in the chiefdoms (sub-districts) targeted for MDA; and of suspected Ebola alerts.

## Methods

### Ethical clearance

The study was approved by the NMCP, Ministry of Health and Sanitation and involved only the use of anonymous aggregated data collected routinely by the health facilities and Ebola alert calls made to the Ebola Command Centres (temporarily established in each district in response to the Ebola outbreak).

The impact of the two MDA rounds was evaluated using reported data on:

1. suspected cases tested with RDT tests, malaria confirmed cases and RDT test positivity rate (TPR) at health facility level.
2. Ebola alerts reported to the Ebola Command Centres.

### Interventions and sites

MDA was implemented in the eight (of the total 14 districts in the country) that are highly endemic to malaria but also were affected by the Ebola outbreak. In six of them, MDA was implemented only in the chiefdoms (the lowest administrative unit) with confirmed Ebola cases. Chiefdoms with no confirmed Ebola case were excluded from the MDA. In the two districts of Western Area (the Capital city, Freetown), however, all zones (equivalent to chiefdoms in the rural areas) except two were covered with MDA in both rounds. The two zones were not covered in the first round of MDA due to stock-outs of ASAQ.

As controls, chiefdoms that did not have confirmed Ebola cases at the time of the MDA, and hence did not receive MDA (non-MDA chiefdoms), were selected from the same districts to ensure that both the MDA- and non-MDA-chiefdoms share similar epidemiological conditions, i.e., similar intensity of malaria transmission and programme coverage.

Coverage and utilization of LLINs was assumed constant throughout the Ebola outbreak as the LLIN mass campaign was conducted nationwide in June 2014, just before the Ebola outbreak [10]. Other factors which may have changed over time during pre- and post-MDA have affected equally the MDA-chiefdoms and non-MDA-chiefdoms in the same districts. Access to RDTs and ASAQs at community level was suspended, but available at health facility level although utilization rates may have varied. In the Ebola holding centres, all suspected cases received full presumptive anti-malarial treatment courses with ASAQ (Additional file 1).

The analysis of impact of MDA on malaria and Ebola-related parameters was made based on comparing the trends of pre- and post-intervention periods.

The coverage of the MDA in each round was calculated as the total number of full ASAQ treatment courses distributed divided by the total number of people targeted for MDA multiplied by 100. Target population was determined based on the Household Registration used for the 2014 LLINs mass campaign [10]. The coverage of MDA under DOT was calculated as the total number of people who took the first dose of the medicine under DOT on the day of visit by the drug distributors divided by total number of people who received full treatment courses multiplied by 100.

### Sampling

Of the 75 health facilities sampled, 65 health facilities had complete data for the analysis. The number of facilities in the non-MDA chiefdoms was limited to 16 because there were only 16 chiefdoms not covered by the MDA. The list of the MDA- and non-MDA-chiefdoms in the MDA targeted districts is shown in Table1.

**Table 1:** List of MDA-chiefdoms and non-MDA-chiefdoms by district

Region	Districts	MDA chiefdoms	Non-MDA chiefdoms (control)
Northern	Bombali	Bombali Shebora, Makari Gbanti, Makeni, Township	Biriwa, Safroko Limba
	Port Loko	Buya Romende, Kaffu Bullom, Koya, Maforki, Marampa, Masimera, BKM	Sanda Magbolonthor, TMS
	Tonkolili	Kholifa Rowalla, Tane	Gbonkolenken, Kholifa Mabang
	Koinadugu	Wara-wara Yagala, Sengbeh, Neine	Wara-wara Bafodia, Kasonko
	Kambia	Mambolo, Samu	Braimaia, Magbema
Southern	Moyamba	Lower Banta, Kori	Kayamba, Fakunya
Western Area	Urban	Western Area Urban Twenty (20) zones	Moyiba (Zone5), Zone11 (both low MDA coverage)
	Rural	Ten (10) zones	Hamilton, Goderich

The inclusion criteria for the sample health facilities were: (1) primary health units (PHUs) providing testing services with RDTs (^®^First Response Pf Pan); (2) hospitals with inpatient and laboratory services (malaria microscopy or RDTs); and (3) viral haemorrhagic fever (VHF) laboratories serving Ebola holding centres and treatment centres. One health centre per chiefdom was selected based on random sampling from the sampling list of functioning health centres with diagnostic services in each chiefdom. Health centres with incomplete data were replaced by other health centres from the same chiefdom.

### Age groups

Data forms were designed to collect data by three age groups, i.e., 6–59 months, 5–13 years and 14+ years old.

### Study period

Data collected covered 8 weeks prior to the 1st round of MDA (as baseline) and 8 weeks post the 2nd round of MDA (2nd week of October 2014 to 1st week of March 2015).

### Representativeness

The case series recorded in the selected health facilities are assumed to represent the population living in the respective catchment area (Chiefdom).

### Reporting completeness and data quality

The daily registers were validated and aggregated to weekly data by field surveyors. The selected facilities had a minimum of 20 complete weekly data out of the total 25 study period weeks for confirmed outpatient malaria cases and inpatient malaria cases.

### Data elements

The following data elements were collected from the sample health facilities:

1. *Outpatient all*-*cause consultations (all*-*cause OPD)*—number of patients visiting out-patient clinic or ward.
2. *Outpatient malaria cases (OPD malaria)*—number of cases registered as either probable (based on clinical presentation in the absence of testing) and cases confirmed with either microscopy or RDT.
3. *Suspected cases tested*—number of suspected cases tested with either malaria microscopy or RDT.
4. *Confirmed malaria cases*—number of positive cases confirmed with either malaria microscopy or RDT.
5. *Inpatient all*-*cause cases (all*-*cause IPD)*—number of patients admitted for different health conditions to the hospital.
6. *Inpatient malaria case (IPD malaria)*—number of severe malaria cases admitted. Most of the inpatient malaria cases are assumed to be confirmed either at the admission or after the patient has been admitted although some may be admitted on clinical basis.
7. *Ebola alert*—number of phone calls received at the 117 free hotline at District Ebola Central Command from individuals with suspected Ebola symptoms.
8. *Ebola suspected case*—any person, alive or dead, suffering or having suffered from a sudden onset of high fever and had contact and or cared for a suspected, probable or confirmed Ebola case, or a dead or sick animal, attended a funeral of someone with Ebola or any person with sudden onset of high fever and at least three of the following symptoms: headache, vomiting, anorexia/loss of appetite, diarrhoea, lethargy, stomach pain, aching muscles or joints, swallowing difficulties, breathing difficulties, or hiccup; or any person with unexplained bleeding OR any sudden, unexplained death [11].
9. *Ebola confirmed case*—a probable case (any suspected case evaluated by a clinician or any deceased suspected case having an epidemiological link with a confirmed case) or a sample from a person tests positive for Ebola virus with polymerase chain reaction (PCR) in the laboratory.
10. *Ebola negative case*—a sample from a probable or suspected person who tests negative for Ebola virus with two consecutive PCR tests in the laboratory.

In addition, the following additional indicators were generated and analyzed:

1. test positivity rate (TRP)—positive malaria RDTs divided by tested cases multiplied by 100,
2. non-malaria outpatients—all-cause outpatient consultations minus confirmed malaria cases,
3. non-malaria admissions—all-cause admissions minus malaria admissions,
4. proportion of outpatient malaria—confirmed outpatient malaria cases divided by all-cause outpatient cases multiplied by 100 and,
5. proportion of malaria admissions—inpatient malaria cases divided by all-cause admissions (inpatient cases) multiplied by 100.

### Data management and statistical methods

An Excel daily data collation tool with automated data merging features and paper forms, consistent with the Excel daily data forms were used for the field data collection. Stata 14 [12] was used to compile data by week to perform statistical analysis. Trends in indicators related to malaria and Ebola alerts were analysed for the pre- and post-MDA periods. The impact during each of the 4 weeks post-MDA was analysed although the impact at the 3rd week after each MDA round is the expected maximal impact considering the half-life of the medicine (amodiaquine). Change in indicators over time (pre- and post-MDA) stratified by MDA and non-MDA chiefdoms were assessed as relative percent change using a segmented (interrupted time series) regression model. The relative percent change was calculated by comparing the slop trend in the pre-MDA weeks to the slop predicted during post-MDA weeks, assuming a continuation of the pre-MDA trend if there were no MDA. The 95 % confidence intervals (CI) around the estimates were computed by using the CIs around the regression coefficient estimates using the delta method [13]. Confidence intervals not including zero value are interpreted as statistically significant change with either decrease (negative intervals) or increase (positive intervals) compared to pre-MDA trends.

The application of the segmented-time series regression model is described in (Additional file 2).

### Comparisons

1. *Pre*-*MDA versus post*-*MDA—changes* comparison of the trends during pre-MDA versus post-MDA periods, stratified by MDA and non-MDA-chiefdoms.
2. *MDA versus non*-*MDA—chiefdoms*—comparison of trends in the MDA-chiefdoms versus the non-MDA-chiefdoms from the same districts.
3. *Non*-*malaria diseases*—comparison of trends of malaria versus non-malaria outpatient cases and admissions during pre-MDA versus post-MDA period in both the MDA- and non-MDA-chiefdoms.

## Results

### Coverage of the intervention (MDA)

**Fig. 1:**
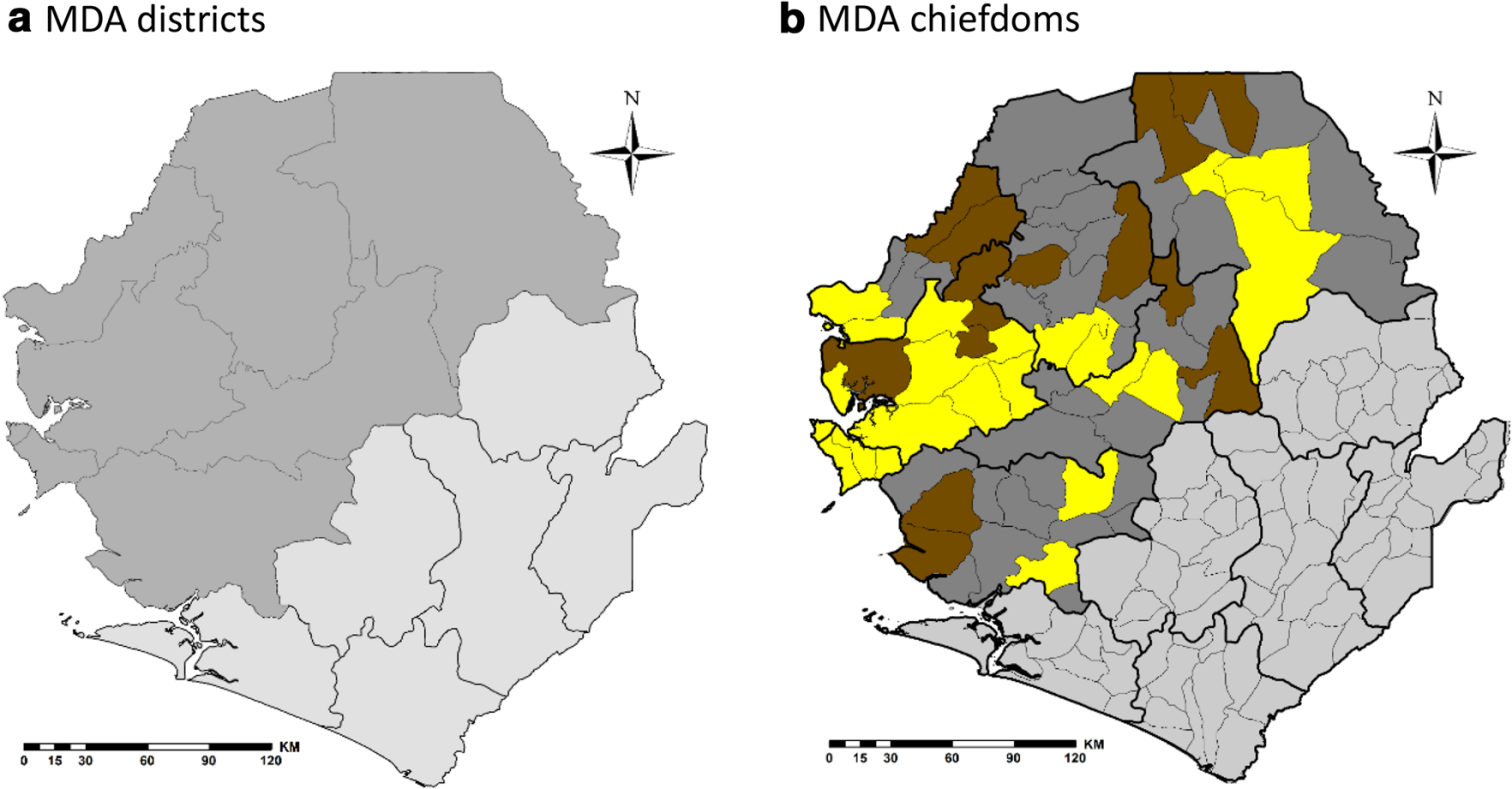
Map of chiefdoms in the eight districts covered by two rounds of MDA with ASAQ. **a** MDA by district (*dark shaded*); **b** MDA by chiefdoms (MDA chiefdoms—*yellow shaded*; and non-MDA chiefdoms—*dark-brown* shaded)

The districts and chiefdoms (within the targeted districts) that were covered with MDA are shown in Fig. 1.

The coverage of the MDA in the targeted 24 chiefdoms in six rural districts; and 30 zones in the Western Area, Freetown (10 zones in the Rural and 20 in Urban areas) was 87 % in the first round and 96 % in the second round (Table 2). The targeted population for Western Area was particularly under estimated due to lack of up-to-date population census-leading to stock out of ASAQ for two zones (Zone 5 and 11 in the urban areas) in the first round. This was less a problem in the rural areas because of the stable population. The coverage of DOTs, measured only in the second round, was 71 % as the data collection forms in the first round did not include DOTs.

**Table 2:** Coverage of two rounds of MDA with ASAQ by district (comparing the total number of full treatment courses distributed with target population) and directly observed treatment (DOTs), measured only in round 2

Round 1						Round 2				Average (two rounds)		
District	MDA Chiefdoms	Total Population	ASAQ distributed	DOT coverage (%)	MDA coverage (%)	Target	ASAQ distributed	DOT coverage (%)	MDA coverage (%)	Population targeted	Population covered	Average MDA coverage (%)
Bombali	Bombali Shebora, Makari Gbanti	244,468	219,468	*–*	95	218,377	199,118	82	91	231,423	209,293	90
Koinadugu	Wara–wara Gagala, Sengbeh, Neini	133,313	130,647	–	98	135,151	133,319	69	99	134,232	131,983	98
Tonkolili	Kholifa Rowala, Tane	123,184	109,634	–	89	128,999	118,815	91	92	126,092	114,225	91
Port Loko	Buya Romende, Kaffu Bullom, Koya, Maforki, Marampa, Masimera, BKM	442,979	349,953	–	79	525,473	447,860	75	85	484,226	398,907	82
Kambia	Mambolo, Samu	119,924	116,326	**–**	97	117,183	109,969	79	94	118,554	113,148	95
Moyamba	Lower Banta, Kori	77,784	69,228	**–**	89	76,228	74,218	97	97	77,006	71,723	93
Western Area Urban	Western Area Urban, 20 zones	1,550,781	1,250,781	**–**	87	1,444,931	1,454,321	64	101	1,497,856	1,352,551	90
Western Area Rural	Western Area Rural, 10 zones	328,463	278,463	**–**	84	393,826	395,843	73	101	361,145	337,153	93
Total		2,899,500	2,524,500	*–*	87	3,043,168	2,933,463	71	96	3,030,532	2,728,982	90

### Impact of the MDA

The impact of the two rounds of the MDA on the malaria burden and suspected Ebola cases measured in the relative percent changes of the trends of key indicators at each of the 4 weeks following the two rounds of the MDA are presented in Table 3. The relative percent changes during the post-MDA period compared to the trends of the pre-MDA period are expressed in percentages with 95 % CI in brackets. Confidence intervals that *exclude zero* are interpreted as statistically significant change (Additional file 3).

**Table 3:** Relative percent change in malaria indicators and Ebola alerts in the health facilities in MDA (n = 34 PHUs) and non-MDA chiefdoms (n = 14 PHUs) during post-MDA weeks using interrupted time-series regression

Chiefdoms	Indicators	Relative percent changes in 1st MDA round (%)					Relative percent changes in 2nd MDA round (%)			
		Weeks post 1st MDA					Week number after second MDA			
		Baseline	1	2	3	4	1	2	3	4
MDA Chiefdoms	Malaria (clinical + confirmed)	603	−45^*^	−43^*^	−42^*^	−10	−36^*^	−20	−3	25
	RDT tested	516	−43^*^	−41^*^	−36^*^	−6	−39^*^	−16	−31^*^	23
	RDT positive	398	−47^*^	−62^*^	−53^*^	−35^*^	−55^*^	−49^*^	−58^*^	−26^*^
	Test positivity rate	77	−6^*^	−35^*^	−25^*^	−29^*^	−22^*^	−35^*^	−34^*^	−34^*^
	Proportion of outpatient malaria (of all-cause outpatient)	28	−33^*^	−57^*^	−44^*^	−33^*^	−52^*^	−60^*^	−67^*^	−46^*^
	Non-malaria	1019	−9	11	2	14	24	77	84	86
	Ebola alerts	324	−30^*^	−12	−23^*^	−18	−48^*^	−59^*^	−58^*^	−60^*^
	Inpatient malaria cases	78	−31^*^	−20^*^	−9	8	−19	−26	−25	−0.6
	Proportion of inpatient malaria (of all-cause inpatients)	35	−24^*^	−16^*^	16	24	−2	−70	−16	28
Non-MDA Chiefdoms	Malaria (clinical + confirmed)	290	−9^*^	−2	−33^*^	−26^*^	−33^*^	−18^*^	−33^*^	−39^*^
	RDT tested	403	−23^*^	−11^*^	−50^*^	−44^*^	−43^*^	−44^*^	−36^*^	−51^*^
	RDT positive	237	−30^*^	−9	−47^*^	−37^*^	−53^*^	−38^*^	−45^*^	−51^*^
	Test positivity rate	59	−8^*^	4	8	16^*^	−14^*^	17	−7	6
	Proportion of outpatient malaria (of all-cause outpatients)	39	−33^*^	−2	−22^*^	−3	−37^*^	−20^*^	−31^*^	−27^*^
	Non-malaria	374	29^*^	−8	−20^*^	−35	−4	−14	−2	−21
	Ebola alerts	198	0.1	2	7^*^	10^*^	−16^*^	−19^*^	−22^*^	−32^*^

Percentages with asterisk are significant changes (95 % CI that excluded zero). Negative changes are decrease and positive changes are increase in trends post-MDA compared to trends of pre-MDA weeks

### Changes in the MDA-chiefdoms during the post-MDA weeks

**Fig. 2:**
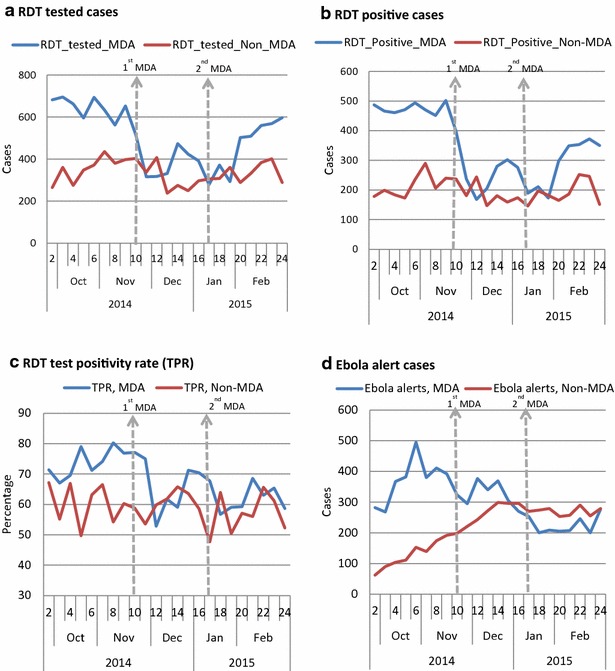
Trends of malaria suspected cases tested with RDT, RDT positive cases, RDT TPR and Ebola alert cases in MDA-chiefdoms (n = 34 PHUs) and non-MDA-chiefdoms (n = 14 PHUs). **a** RDT tested cases. **b** RDT positive cases. **c** RDT test positivity rate (TPR). **d** Ebola alert cases. *Dotted vertical lines* indicate the start of 1st MDA (week 11) and 2nd MDA (week 17)

**Fig. 3:**
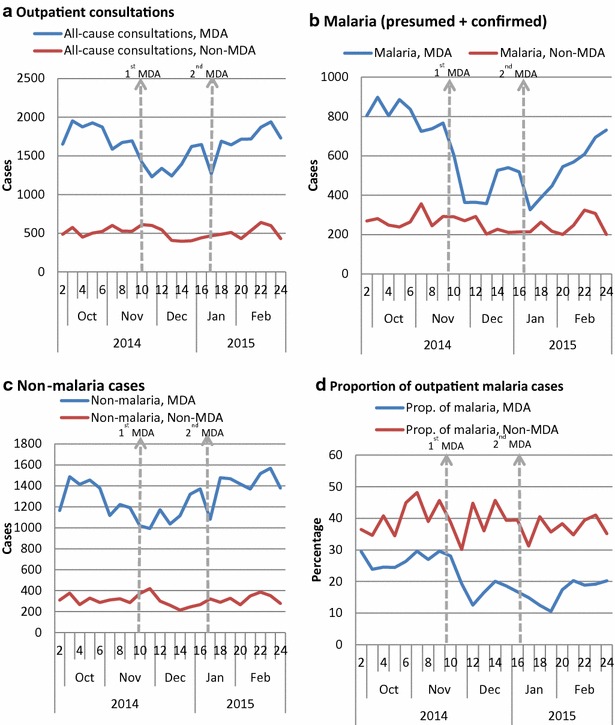
Observed trends of malaria indicators in primary health units (PHUs) of MDA-chiefdoms (n = 34 PHUs) and non-MDA-chiefdoms (n = 14 PHUs). **a** OPD cases. **b** Malaria (presumed + confirmed). **c** Non-malaria cases. **d** Proportion of malaria of all OPD cases. *Dotted vertical lines* indicate the start of 1st MDA (week 11) and 2nd MDA (week 17)

After two rounds of the MDA, the number of suspected cases tested with RDT decreased significantly by 43 % (95 % CI 38–48 %) at week 1 and remained significantly low at week 2 and 3 post-first MDA and at week 1 and 3 post-second MDA; RDT positive cases decreased significantly by 47 % (41–52 %) at week 1 and remained lower at all the weeks post-first and second MDA; RDT test positivity rate (TPR) declined by 35 % (32–38 %) at week 2 and stayed significantly low at all the weeks of post-first and second MDA (Table 3; Fig. 2a–c). Confidence intervals are shown in Additional file 3. The total malaria (clinical + confirmed) cases decreased significantly by 45 % (39–52 %) at week 1 and were lower at week 2 and 3 after the first MDA; and week 1 after the second MDA. The proportion of confirmed malaria cases (of all-outpatients) fell by 33 % (29–38 %) during all the 4 weeks of post-first and second MDA. On the contrary the non-malaria outpatient cases (cases due to other health conditions) either remained unchanged or fluctuated insignificantly during the observation weeks (Table 3; Fig. 3a–d).

The Ebola alerts (117 hotline calls) in the District Ebola Command Centres decreased significantly by 30 % (13–46 %) at week 1 after the first MDA and by much lower during all the weeks after the second MDA (Table 3; Fig. 2d and Additional file 3).

The number of malaria inpatient cases decreased significantly by 31 % (22–39 %) and 20 % (7–32 %) at week 1 and 2 post-first MDA respectively. The proportion of inpatient malaria (of all inpatients) fell by 24 % (12–35 %) at week 1 and by 16 % (0.4–31 %) at week 2 post-first MDA. The total inpatient cases in the hospitals did not change during the same period (Table 3) except at week 3 post-first MDA.

### Changes in the non-MDA-chiefdoms during the post-MDA weeks

The number of suspected cases tested with RDT decreased by 23 % (17–30 %) at week 1 and remained low at all the weeks after the first and second MDA; the number of RDT positive cases decreased by 30 % (22–38 %) during week 1 post-first MDA and stayed low throughout all the post-MDA weeks (except week 2 post-first MDA); the decrease in RDT test positivity rate (TPR) was low but significant at week 1 and 4 of post-first MDA and week 1 of second MDA (Table 3 and Fig. 2a–c). The total malaria (clinical + confirmed) cases decreased significantly in all the weeks post-first and second MDA except the second week post-first MDA; the proportion of confirmed malaria cases (of all-cause outpatient cases) fell by 33 % (25–40 %) at week 1 and stayed low at week 3 post-first MDA and all weeks post-second MDA. The changes in the non-malaria outpatient cases during the first MDA weeks fluctuated inconsistently (Table 3; Fig. 3a–d) but were insignificant throughout all the post-second MDA weeks.

Predicted Ebola alerts (117 hotline calls) in the non-MDA-chiefdoms showed an increase of 7 % (3–11 %) and 10 % (6–15 %) at week 3 and 4 respectively post-first MDA but remained significantly lower throughout the second post-MDA weeks although the observed values showed increasing trend starting the second week of post-second MDA (Table 3; Fig. 2d).

The trend of inpatient malaria cases in the non-MDA-chiefdoms was omitted from the analysis owing to inadequate hospital inpatient data (only one hospital).

Microscopic test results from the hospitals were excluded from the analysis due to incompleteness and inconsistency of the data as only few hospitals were conducting microscopy during the Ebola epidemic. RDT records in the PHUs were complete for all-ages only, therefore, age-specific data analysis was omitted from the results. The data on parasitological confirmation of malaria in the VHF laboratories was incomplete and excluded from the analysis as most laboratories started confirmation of malaria with RDT after the second MDA was conducted.

**Fig. 4:**
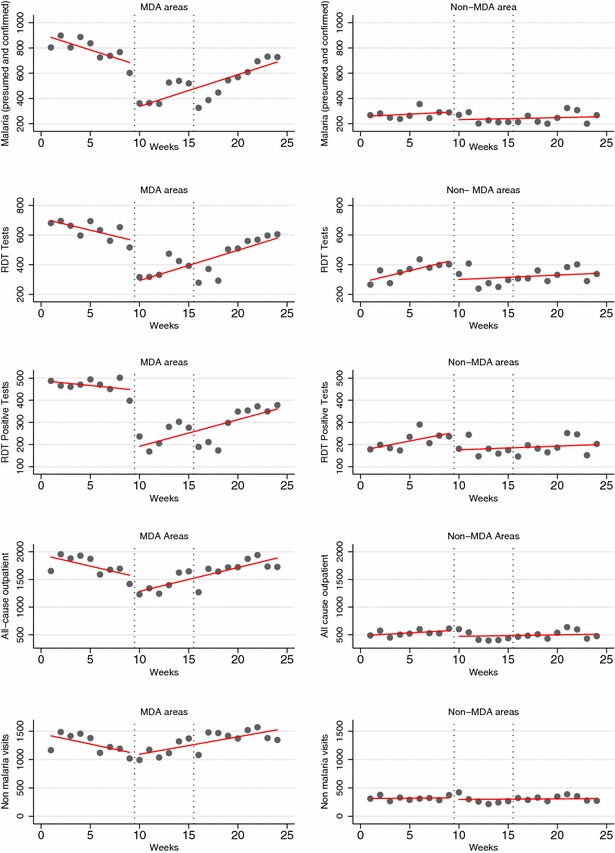
Regression of trends of malaria indicators during pre-MDA and post-MDA weeks in MDA-chiefdoms (n = 34 PHUs) and non-MDA-chiefdoms or controls (n = 14 PHUs). *Dotted vertical line* shows the start of the first MDA (week 11)

Figure 4 summarizes the regression trends of malaria indicators during the pre-MDA and post-MDA weeks in the MDA and non-MDA chiefdoms indicating marked break points of the pre- and post-MDA slopes in the MDA chiefdoms compared to the non-MDA chiefdoms.

In summary, the results show significant decrease in the key malaria indicators including suspected cases tested with RDT, confirmed cases, test positivity rates and proportion of outpatient consultations due to malaria during the post-MDA period compared to the pre-MDA period. Non-malaria cases remained relatively stable or increased during the post-MDA periods in the MDA-chiefdoms. The number of inpatient malaria cases also decreased moderately although statistically insignificant. The Ebola alerts decreased significantly after the 2nd round of the MDA.

However, significant reduction in trends of malaria indicators also occurred in the non-MDA-chiefdoms (controls) although the decreases were much stronger in the MDA-chiefdoms throughout all weeks of the two rounds of MDA.

## Discussion

The results of the study show the impact of the MDA implemented in-line with the recent WHO recommendations as emergency response in the Ebola affected countries. All logistic aspects, community and social mobilization efforts were addressed and put in place in less than 2 months. The two rounds of the MDA were given to >90 % of the population, out of which 71 % received the anti-malarial medicines under directly-observed treatment. The operational coverage reported by the NMCP during MDA is very similar to the results of the Independent Monitors of the MDA, a national survey team deployed by the MoHS immediately after the MDA. The monitors reported >90 % household visits by drug distributors; >75 % compliance in taking full treatment courses; and >74 % DOTs [14]. Assuming the 71 % DOTs coverage measured on the 1st dose of the treatment course would be similar in all other treatment days of the 1st and 2nd round and assuming a similar adherence to treatment among the people who received the anti-malarials without DOTs, this means that at least 60 % of the population received full effective coverage (i.e. 0.90 x 0.71 = 60 %) with artesunate + amodiaquine. Considering the timing of adoption and implementation; and the complexity of the operation amidst of the Ebola epidemic (high apprehension), attaining such a moderate effective coverage rate of MDA (covering >2.7 million people on average in two rounds) is a remarkable achievement. Household surveys on the adverse drug reaction (ADR) during the MDA by teams of health workers trained in pharmacovigilance reported common adverse drug reactions already described in the product information leaflet namely: weakness, dizziness, headaches and diarrhoea. But no other unexpected event was reported. A few deaths suspected to be associated with the MDA were ruled out after thorough investigations [15].

The impact of the MDA was confirmed by the sharp and significant reduction of the suspected malaria cases that were tested with RDT (>40 % decrease) immediately after the 1st round of MDA signifying a decrease in the febrile cases that would have visited the primary health units (PHUs). The number of RDT confirmed cases also declined significantly following the trend of the suspected cases tested with RDT. Other indicators including total malaria cases (clinical and confirmed) and proportion of the malaria outpatients followed the same pattern. The trend of other disease conditions (non-malaria) was stable or increasing and as expected not influenced by the MDA with ASAQ. The significant decrease in the number of RDT tests and RDT confirmed cases in the non-MDA-chiefdoms may be the result of cross-over of people from non-MDA-chiefdoms to the MDA-chiefdoms to benefit from the MDA. Such high population’s demand for medications is expected owing to the apprehension caused by the Ebola epidemic and is consistent to the report of the MDA implementation by the MoHS which stated that such movement could be one of the main reasons for stock-outs of ASAQ in the Western Area, Freetown during the first MDA [16].

The significant reduction in the suspected malaria cases tested could in principle also result in a reduction of suspected Ebola cases owing to the close similarities of clinical signs of both diseases. The benefit of the MDA as a temporary intervention was to offer treatment to a large number of malaria cases that would have been left untreated at home; transient prophylaxis to potential malaria cases; and in reducing patient case-load in the severely strained health facilities. In addition, as the timing of the MDA campaign coincided with the peak of the Ebola epidemic and high malaria transmission season, the number of Ebola nosocomial cases and burden of malaria averted could be remarkably high owing to the significant decrease in the number of suspected malaria cases (febrile cases) and reduced number of Ebola alerts in the MDA-chiefdoms. The impact of the MDA on malaria deaths could not be measured in this study as all deaths, both at the service units or community level, were handled by the Safe Burial Teams regardless of their causes (Additional file 1). Nevertheless, a reduction in malaria-specific mortality proportional to the reduction of confirmed malaria cases could be expected (assuming the same case fatality rate).

The impact of the MDA on the reduction of the clinical malaria waned nearly after the 4th week of each MDA round and malaria trends returned nearly to the pre-MDA levels coinciding with the expected maximum half-life of 240 h (10 days) of amodiaquine [17]. The return of malaria to pre-intervention levels after 4 weeks of MDA is most likely due to re-infection of the population owing to high entomological inoculation levels as amodiaquine resistance has not yet been reported in the country [18]. Therefore, reduced level of malaria infection cannot be sustained for long with MDA in high transmission setting using an anti-malarial drug with moderate half-life unless there is a simultaneous reduction in the vectorial capacity. Consistent to results of previous studies, there was no effect on the malaria transmission [19]. Since ASAQ has a relatively moderate half-life and no gametocytocidal drug (primaquine) was used in the campaign, no impact on long-term reduction of malaria transmission was expected. Nonetheless, in this unprecedented effort covering a very large population, the number of malaria and fever cases (potential nosocomial infections) were significantly reduced as per the intention of MDA campaign. The effect documented in this study confirms the intention of the intervention to create a temporary reduction in malaria burden. A more sustained reduction of infection would require an anti-malarial medicine with longer half-life complemented with other aggressive interventions aiming at reduction of the vectorial capacity.

The study had the following limitations: (1) reduced treatment seeking behaviour and testing patterns due to the effects of Ebola epidemic as the dynamics were changing over time although this had stabilized prior to the first MDA; (2) inability to properly estimate the population size (particularly in the urban districts of Western Area) leading to shortage of medicines during the first round of MDA in two zones of Freetown which had lower MDA coverage; (3) cross-over of populations from non-MDA to the MDA-chiefdoms during the campaign to access treatment may be the reason for the little difference in the effect of the MDA between the MDA- and non-MDA-chiefdoms (Table 3); (4) implementation of DOTs was not measured in the first round although this was rectified in the second round of MDA; (5) the Ebola holding centres had limited testing to Ebola only at the VHF laboratories (no systematic parasitological testing for malaria). All suspected cases in the Ebola holding centres were treated presumptively with first-line anti-malarial treatment without RDT testing (although some started testing and recording only after the 2nd MDA); (6) persistence of antigenaemia for *Plasmodium falciparum* histidine-rich protein 2 (*Pf*HRP2), such as those used in Sierra Leone, after treatment or recovery from infection (RDTs false positive) for about 40 days [20] may have led to underestimation of the impact of MDA on reducing the test positivity rate. Duration of persistent positivity is very long with HRP2 when compared with microscopy as gold standard; and short when compared with PCR [21]. Therefore, the value of using RDTs in evaluating impact of MDA on transmission reduction in different transmission intensity merit further studies.

## Conclusion

The MDA with first-line ACT, given to more than a third of the population of Sierra Leone as a temporary measure in response to the Ebola outbreak at moderate levels of effective coverage, has resulted in significant reduction of malaria morbidity and of suspected malaria cases that would have led to Ebola nosocomial infections. The intervention also helped reduce outpatient case-load to the strained health system at the peak of the Ebola outbreak.

As expected, the effect of the MDA on malaria burden and febrile cases waned quickly and malaria transmission returned nearly to the pre-MDA levels in about 4 weeks. Consistent to other studies, the intervention showed a relatively short-lived impact of MDA in high transmission areas when deploying medicines with moderate half-life. From the outset, long-term impact on reduction of malaria transmission was not the goal. Nonetheless, the approach was an appropriate public health intervention in the context of the Ebola epidemic even in high malaria transmission areas of Sierra Leone. The deployment of MDA using medicine with moderate half-life in areas of high transmission in situations other than complex emergencies like the Ebola epidemic in Sierra Leone would not be effective.

## Additional files

10.1186/s12936-016-1493-1 Diagrammatic presentation of Ebola-Malaria algorithms in Sierra Leone, 2014–2015.
10.1186/s12936-016-1493-1 Diagrammatic design of the interrupted time–series regression used to measure impact of the MDA.
10.1186/s12936-016-1493-1 Relative percent change in malaria indicators and Ebola alerts in the health facilities in MDA- (n = 34 PHUs) and non-MDA-chiefdoms (n = 14 PHUs) during post-MDA weeks using interrupted time-series regression. Upper values are relative percent changes and lower values in brackets are 95 % CI. Percentages in bold are significant changes with 95 % CI that excluded zero. Negative changes are decrease and positive changes are increase in trends post-MDA compared to trends of pre-MDA weeks.

## Abbreviations

ACT: artemisinin-based combination therapy
ASAQ: artesunate-amodiaquine
CI: confidence interval
DOTs: directly-observed treatment
EVD: Ebola viral disease
LLIN: long-lasting insecticidal net
MDA: Mass Drug Administration
MDA-chiefdoms: chiefdoms that received MDA
MoHS: Ministry of Health and Sanitation
NMCP: National Malaria Control Programme
Non-MDA-chiefdoms: chiefdoms that did not receive MDA
PHUs: primary health units
PCR: polymerase chain reaction
RDT: rapid diagnostic test
TPR: test positivity rate
WHO: World Health Organization
