# Supplementary figures and images for: Impact of the Mass Drug Administration for malaria in response to the Ebola outbreak in Sierra Leone
Source: Malar J. 2016 Sep 20;15:480. doi: 10.1186/s12936-016-1493-1 (PMC5028945; doi:10.1186/s12936-016-1493-1)

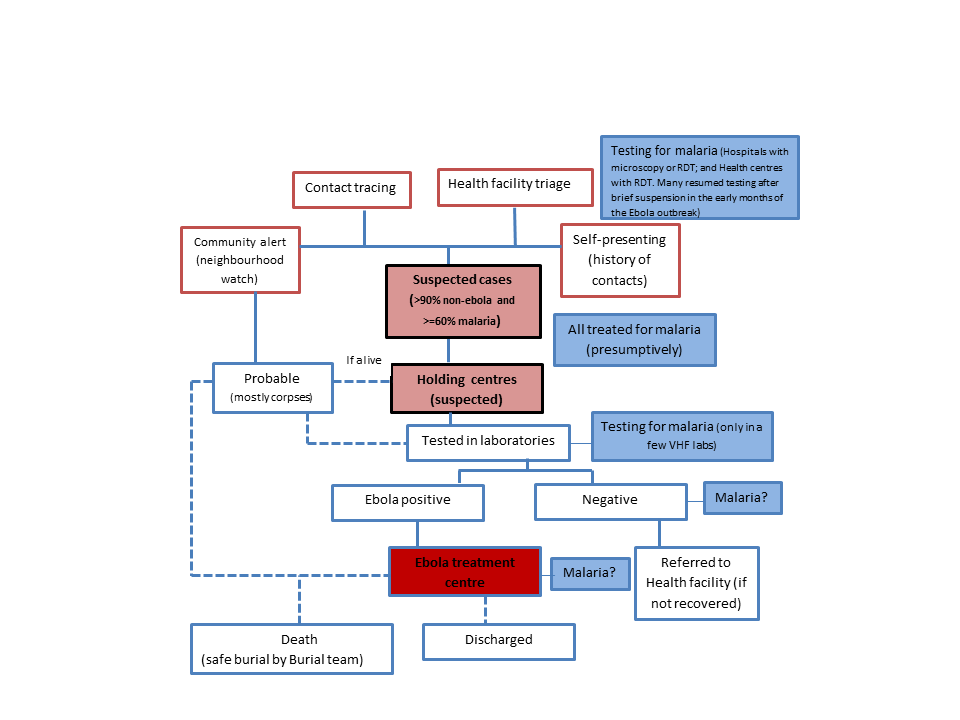

Supplement: Supplementary file 1 — 10.1186/s12936-016-1493-1 Diagrammatic presentation of Ebola-Malaria algorithms in Sierra Leone, 2014–2015. [file 12936_2016_1493_MOESM1_ESM.tif]

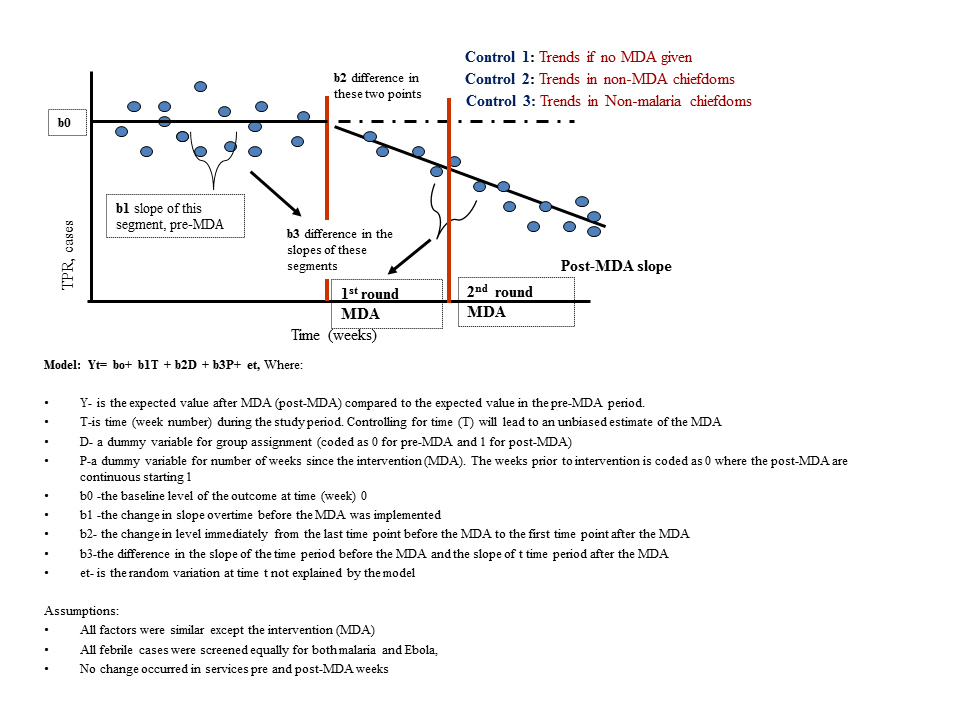

Supplement: Supplementary file 2 — 10.1186/s12936-016-1493-1 Diagrammatic design of the interrupted time–series regression used to measure impact of the MDA. [file 12936_2016_1493_MOESM2_ESM.tif]
